# Finishing the Plate

**Published:** 1907-02

**Authors:** Stewart J. Spence

**Affiliations:** Chattanooga, Tenn.


### The American Journal of Dental Science

The Official Organ of the Florida, Georgia and South Carolina State Dental Societies

**Third Series**

VOL. XXXVIII    FEBRUARY, 1907    NO. 2

EDITORS

WM. GIRD BEECROFT, D. D. S., Madison. Wis.    B. H. TEAGUE, D. D. S., Aiken, S. C.

Published on the 10th day of each month.
Subscription price $1.00 per year.
ENTERED AS SECOND-CLASS MATTER, MADISON, WIS.

Address all communications, both business and editorial, to
**The Dental Publishing Company, Madison, Wisconsin**

### FINISHING THE PLATE

STEWART J. SPENCE, CHATTANOOGA, TENN.

Having vulcanized the piece, do not cool it rapidly if the investment has been of plaster of Paris, because sudden cooling tends to warping. The writer once caused a vulcanite plate to crack severely by rapid cooling, the investment having been of plaster of Paris hurried into the vulcanizer soon after mixing.

Having opened the flask, brush away the plaster adhering to the plate, or if it is very adherent, immerse it for ten or fifteen minutes in fifty per cent aqueous solution of sulphuric acid. This will soften the plaster so that it may easily be brushed away, and has no injurious effect on the vulcanite. Hydrochloric acid, however, rapidly dissolves aluminum.

Reduce the plate with file and chisel, and either with scraper or revolving sandpaper. I prefer the latter. No. 0 sandpaper is best, for anything coarser makes scratches in the vulcanite which are not easily removed with the pumice. For polishing the interdental vulcanite a revolving brush with pumice is best, and the coarse bristle brushes are much better than the soft, because more penetrating, reaching in between teeth.

Do not rashly reduce the borders of the plate. Reduction can be made later if necessary. The farther these borders extend the better they exclude air and food from getting under the plate; and, in the lower plate, the greater the surface the less the liability to soreness or pain under the pressure of mastication; just as a large saddle is less hurtsome to a horse's back than a small one. I do not favor the theory that we ought to "cut away the borders of the lower plate till we feel sure we have cut as much as we dare, and then cut away a little more." On the contrary, I regard it better to let a lower plate impinge a little on the muscles at first, rather than make it so small as to be forever a source of discomfort under pressure of mastication. The muscles will yield to it in the course of time. It is, however, quite important to reduce the border which in the lower plate lies bucally to the last molar, which if too far extended interferes with the muscles of mastication. Also a lower plate must not extend posteriorly over a soft membrane, because the opening of the mouth will by stretching this membrane, raise the plate at its "heels." It must also be quite free at the *franum lingua;* which does not yield to pressure.

On placing the teeth in the mouth test the correctness of occlusion by asking the patient to shut the jaws and then very slightly open them and shut again, repeating this several times, until he can say if one side occludes earlier than another. Or, soften a piece of baseplate wax, insert it in mouth, and let patient close on it. If some point or points occlude too early, detect them by carbon paper (the thin is best) and grind accordingly.

Next ask patient to make the grinding bite slowly, and see if the teeth occlude properly during it. Do this on both sides. The occlusion of the molars is, of course, hidden by the cheeks, but that of the five anterior teeth (from the second bicuspid forward) can usually be observed. If one of these is found a little too long for the rest, grind it down. In this grinding bite the posterior molars ought to occlude on one side when the cuspids occlude on the other. This, however, is often not possible where some of the patient's natural teeth remain.

As regards adaptation: place the plate in the mouth and with one finger on the left molars and one on the right press heavily first on one side then the other. Do similarly with posterior and anterior regions. If you get much sound of air escaping under this pressure your plate probobaly rocks on the palate. This is the common form of misfit, and a very bad one. Even if its adaptation to the palate gives considerable resistance to downward traction such a plate will be much less stable during mastication than one which presses on the alveolar ridge rather than the palate. This pressure on palate results from warpage or from the expansion of model. It may be remedied by immersing the plate in hot water, which reduces its size and also restores it to its original shape,—that which it had before being warped in cooling. There is danger, however, of reducing the plate too much, and it is well to begin by immersing it in water at about 160 degrees F. for a few minutes, then trying in the mouth, and raising the temperature till best results are reached; which in extreme cases may need water at 212 degrees F. Use a thermometer in the water. The plate need only be immersed long enough to be heated through. Such a process for reduction is never needed if Spence's Plaster is used for model, for no expansion will exist; nor for warpage, if it be used for inyestment, because its hardness will not allow the plate to warp.

Caution the patient that he is apt to have his patience tried in his first efforts at eating, and recommend that he practice between meals by chewing something which may be masticated without swallowing, such as chewing gum, wax, a clean rag, etc.

Advise the patient to rest the mucous membrane by leaving his plate out at night, placing it in water to prevent mucus drying on it, with a little common salt in the water to destroy the microbes which adhere to the mucus.

This concludes my series of papers on prosthesis. I have confined them to platework, and chiefly to vulcanite, because metal plate-work and bridge-work would have opened up too wide a field.

I hope my readers will not censure me for the frequent allusions to my plaster, for I could not have omitted this mention without withholding what I consider to be the true and correct instructions.

